# Nontrombotic Pulmonary Embolism: Different Etiology, Same Significant Consequences

**DOI:** 10.3390/jpm13020202

**Published:** 2023-01-23

**Authors:** Oana Sirbu, Victorita Sorodoc, Mariana Floria, Cristian Statescu, Radu Sascau, Catalina Lionte, Ovidiu Rusalim Petris, Raluca Ecaterina Haliga, Paula Cristina Morariu, Andreea Tirnoveanu, Vladut Mirel Burduloi, Corina Ursulescu, Laurentiu Sorodoc

**Affiliations:** 1 Department of Internal Medicine, Clinical Emergency Hospital “Sfântul Spiridon”, 700111 Iasi, Romania; 2 Faculty of General Medicine, University of Medicine and Pharmacy “Grigore T. Popa”, 16 Universitatii Street, 700115 Iasi, Romania; 3 Department of Cardiology, Institute of Cardiovascular Diseases “Dr. George I.M. Georgescu”, 700503 Iasi, Romania; 4 Department of Radiology, Clinical Emergency Hospital “Sfântul Spiridon”, 700111 Iasi, Romania

**Keywords:** nontrombotic pulmonary embolism, gas embolism, fat embolism, tumor embolism, amniotic liquid embolism, septic embolism

## Abstract

Nontrombotic pulmonary embolism represents the embolization of different types of materials (cells, organisms, gas, foreign material) into pulmonary circulation. The disease is uncommon, and clinical presentation together with laboratory findings are nonspecific. Its pathology is usually misdiagnosed based on imaging findings as pulmonary thromboembolism, but the correct diagnosis is essential because different therapeutic approaches are required. In this context, knowledge of the risk factors associated with nontrombotic pulmonary embolism and specific clinical symptoms is fundamental. Our objective was to discuss the specific features of the most common etiologies of nontrombotic pulmonary embolism, gas, fat, amniotic fluid, sepsis and tumors, to provide assistance for a rapid and correct diagnosis. Because the most common etiologies are iatrogenic, knowledge of the risk factors could be an important tool for prevention or rapid treatment if the disease develops during different procedures. The diagnosis of nontrombotic pulmonary embolisms represent a laborious challenge, and endeavors should be made to prevent development and increase awareness of this disease.

## 1. Introduction

Nontrombotic pulmonary embolism (NTPE) represents the embolization of nontrombotic material into pulmonary circulation that can be produced by different cell types (amniotic, adipocytes, tumor, hematopoietic or trophoblastic), organisms (bacteria, fungi), gas or foreign material (catheters, intraoperative materials) [1]. The pathogenesis is related to the underlying embolic agent and is more complex than mechanical vascular obstruction [2]. This obstructive pathology that involves the macro and micro pulmonary circulation may be associated with endothelial and parenchymal injury with a systemic inflammatory reaction [3].

Mechanisms of pulmonary obstruction are diverse. In cases of a foreign body and in the majority of cases with nonbiological material, the mechanism is mechanical obstruction. In most cases of endogenous material or biological products, the deterioration of cardiac, vascular, and pulmonary function can be detected, secondary to inflammatory cascade initiation [4].

Unlike pulmonary thromboembolism, NTPE is uncommon, the incidence varies among studies and by underlying cause [2]. Clinical and laboratory findings of NTPE are non-specific and are often overlooked, with patients being misdiagnosed as having a pulmonary embolism on imaging findings [5]. Usually, the chest CT is the investigation that can detect NTPE, with some specific findings depending on etiology [2]. The diagnosis of this spectrum of diseases relies on knowledge of their parenchymal appearance and relevant clinical history [6].

However, it is an under-evaluated cause of pulmonary embolism that requires a different therapeutic approach, and thus, the knowledge of patients at a high risk and clinical symptoms suggestive of this pathology is very important for differential diagnoses. The typical images of the various nontrombotic agents that embolize to the pulmonary arteries in the relevant clinical context can establish the correct diagnosis [2].

Various causes of NTPE are found in the medical literature (Table 1), but the most common are represented by gas, fat, amniotic fluid, septic and tumoral pulmonary embolisms. In this paper, we try to assess the specificity and the diagnostic possibilities of the most common forms of NTPE to facilitate the critical thinking of different patients.

## 2. Gas Pulmonary Embolism

Pulmonary embolism caused by gas is usually an iatrogenic complication of medical procedures when gas enters the body trough venous access, traveling through the heart into the pulmonary artery. Although air is the main etiology of gas embolism, other gases used in medical procedures can provoke these symptoms, such as carbon dioxide, nitrogen, helium and nitrous oxide [19,20].

The insertion and removal of a central venous catheter may cause a small volume of air to enter the venous circulation, making this procedure a high-risk factor for air pulmonary embolism with an incidence higher than 25%. It is difficult to determine the exact incidence since most cases are not reported because of subclinical symptoms, without severe repercussions [21]. In patients undergoing the intravenous administration of contrast material for computer tomography (CT), a small amount of air can be detected in large intrathoracic veins in 23% of the patients and in 1/47–1/3000 patients after the insertion of central or subclavian catheters [22].

A study on a large series of 677 patients undergoing contrast-enhanced electron beam CT, found minor air embolization in 12% of the patients, with the most common sites being the main pulmonary artery in 8% of the patients [23]. The long-term data in a center specialized in treating venous air embolism has shown a 25% mortality rate, with approximately 50% of the survivors having remaining neurological sequelae [24].

### 2.1. Etiology

Air pulmonary embolism (APE) can occur in several medical situations, including chest trauma and contrast injection during radiographic imaging, such as angiography, pneumocardiographic procedures or needle biopsy [23,25]. Placement, the use and removal of a catheter or other devices, can determine APE with an incidence greater than 25% [21]. Pacemaker implantation, such as an implantable cardioverter defibrillator or cardiac resynchronization therapy, could cause gas embolism in rare causes [26]. Other causes of air embolism are represented by ventilation-induced lung trauma, surgery, gynecological interventions, labor, delivery, lung biopsy, fibrinolytic pleural lavage, laparoscopic abdominal insufflation, which can lead to air, carbon dioxide or argon embolism [18], intra-aortic balloon rupture, barotrauma, arthroscopy, prostatectomy, carotid endarterectomy [1], insufflation of air during endoscopy and the intraoperative use of hydrogen peroxide [7]. Air embolism after endoscopic retrograde cholangiopancreatography is an uncommon complication but it can be determined based on mucosal damage from high-pressure air insufflation, blunt abdominal trauma or biliary venous fistulas [27]. The list of risk factors for gas embolism are presented in Table 2. Awareness of the possible complications of some invasive procedures could prevent or reduce the risk of unwanted outcomes [28]. Moreover, specific symptoms that appear after this type of procedure could increase the suspicion of APE and may facilitate the early initiation of specific treatment.

### 2.2. Pathophysiology

Three elements are required to generate venous air embolism: a source of air, usually atmospheric air, the connection with a vein (for example through a catheter) and a pressure gradient that supports the migration of air into the vein. This gradient is settled when the central venous pressure is lower than the pressure of the atmosphere, for example with deep inspiration, hypovolemia and an upright posture (change in hydrostatic pressure into the venous system because of gravity) [24]. There are some risk factors that could increase the appearance of APE secondary to catheter implantation. Coughing and deep breathing may lead to a lower central venous pressure followed by rapid decompression that would drive the gas into central venous system. Elderly patients (older than 60) are predisposed to air embolism because of their poor tolerance and week activity, which could lead to coughing and because they like high pillows. In a lying position, the pulmonary artery is kept at a high level and once the air enters the circulation, it is settled to remain around pulmonary artery. Other risk factors could be represented by a sheath with a large cavity, the use of sedative drugs, compliance of pulmonary circulation and right heart and a long operating time [26].

The volume of air introduced intravascularly, the velocity and the route of infusion (arterial or venous) will determine the consequences. The mortality and morbidity of APE are associated with the volume of air entered into circulation and the agility of accumulation. In experimental studies, it was concluded that the lethal air volume is approximately 7.5–15 mL/kg for dogs and 0.5–0.75 mL/kg for rabbits [21]. For humans, there was a translation of these results based on case reports from the clinical literature. The lethal volume of air for adults is between 200 and 300 mL or 3–5 mL/kg, but when the air infusion is slow, the heart cand tolerate even larger amounts of air for a long period of time. It has been revealed that at a pressure of 5 cm H_2_O with a 14-gauge needle can transmit 100 mL of air per second. A 5 mm introducer used for the insertion of a hemodialysis catheter can allow approximately 300 mL air to infiltrate the vascular system in 0.5 s [21]. Large volumes of air infused rapidly are most likely to cause major hemodynamic compromise, acute cor pulmonale or asystole; however, even small amounts of air of 100 mL have caused death [22]. The extent of damage determined by APE depends on the volume of gas and the moving speed. The air bubbles from the microcirculation can be dissipated by the solution of air into the blood. If the rate of filtering is overwhelmed by the rate of air influx, discharge into the arterial circulation could occur and ischemic end-organ damage could be determined [24].

Pathophysiologically, the presence of massive quantities of air in the blood stream could lead to obstruction of the right ventricular outflow tract, hampering right ventricular ejection and causing acute dilation of the right ventricle and heart failure. A decrease in right ventricular performance and interventricular septal deviation could lead to a decrease in left heart output and systemic cardiovascular collapse. In the pulmonary capillary beds, activated neutrophils release leukotrienes and thromboxane, which results in an increase in alveolar capillary permeability and airway resistance. These changes cause pulmonary edema and alveolar collapse [20].

The infusion of a smaller amount of air permits migration into the pulmonary artery, causing increased pulmonary pressure, reducing systemic vascular resistance and resulting in a compensatory increase in cardiac output. In response to the presence of air bubbles in the pulmonary arterioles, pulmonary arterial hypertension will be exacerbated by excessive pulmonary vasoconstriction [24].

The acute increase in pressure in the right atrium might cause the air to cross the patent foramen ovale or the atrial septal defect and from the left atrium and left ventricle to ascent into the brain where the consequences could be disastrous, such as an acute cerebral embolism. Paradoxical embolization could also be caused by the presence of pulmonary arteriovenous malformations [26].

The gas entering the pulmonary circulation is atmospheric containing less oxygen and will lead to a decline in blood oxygen content and blood flow. It will cause acute disarrangement of the pulmonary ventilation/perfusion ratio and functional dead space that will worsen the circulatory state. Acute enlargement of the right ventricle will lead to paradoxical movement of the interventricular septum affecting left ventricular diastole [26].

### 2.3. Diagnosis

Air pulmonary embolism should be suspected when patients experience the abrupt onset of respiratory distress in the context of a known risk factor (e.g., intravenous catheter insertion). The findings depend on the severity of pulmonary embolism. If the embolism is minor, the patient could be asymptomatic or experience nonspecific symptoms, such as dyspnea, chest pain, cough, headache, blurred vision and dizziness. Tachycardia, tachypnea and hypotension are common on physical examination and a “mill-wheel murmur” is described occasionally on cardiac auscultation, or a sudden drop in end tidal CO_2_ may be assessed during monitorization [22,23]. Neurologic symptoms, such as altered mental status, seizures and coma, occur in 42% of patients [22]. Any sudden respiratory, cardiovascular or neurologic symptoms after high-risk procedures should indicate the suspicion of pulmonary air embolism [28].

**Figure 1:**
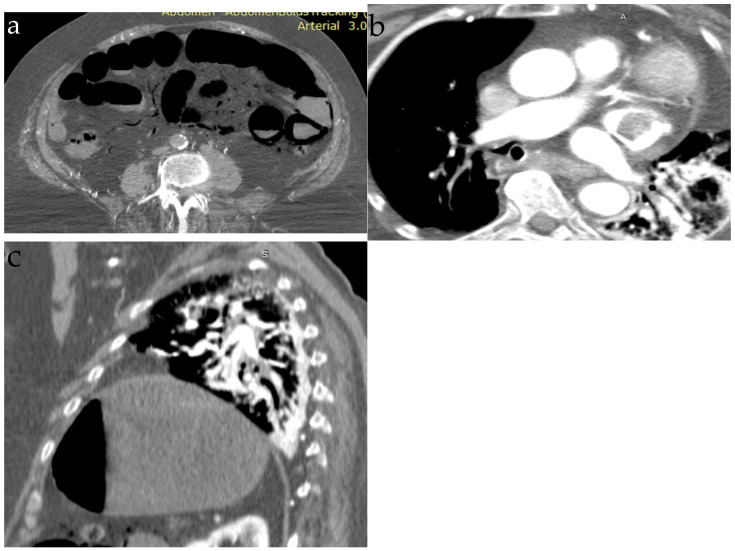
Thoraco-abdominal CT; (**a**) arterial acquisition with air within the branches of the superior mesenteric artery; (**b**,**c**) segmental pulmonary embolism, nonspecific (from the collection of dr. Fotea Vasile).

The diagnosis should be made based on specific findings with different types of chest imaging. On chest x-ray areas of hyperlucency in the main pulmonary artery, the heart or hepatic veins can be observed. Other findings include pulmonary edema and focal pulmonary oligemia. On CT, small amounts of air in the main pulmonary artery, on the right heart or in systemic veins could be detected, but the findings are nonspecific [1]. An example of CT images is presented in Figure 1. In severe cases, pulmonary hypertension and right heart strain could be found [29].

A change in the relationship between ventilation and perfusion is suggested by a decrease in end-tidal carbon dioxide levels, determined via capnometry. The duration and magnitude of the end-tidal carbon dioxide decrease is correlated with the volume of embolized air [18].

Precordial Doppler ultrasound is a good method for detecting intracardiac air, typically used during surgical procedures. Echocardiography is a more sensitive method to detect intracardiac air and patent foramen ovale [30].

Because none of the imaging technique alone are specific enough to be used for diagnosis purposes, clinical suspicion is essential for the initiation of treatment in the context of known risk factors [18]. A tool to make the diagnosis easy is a good understanding of the specific findings for each type of NTPE, which can be found in Table 2.

### 2.4. Treatment

Prevention of air pulmonary embolism is achieved by detecting and removing the risk factors as soon as possible. Gas will continue to enter circulation if risk factors are not eliminated, producing fatal damage to the patient [26]. The prevention of air pulmonary embolism must be focused on training the medical personnel to prevent this avoidable complication [23]. This can be achieved via the detection and removal of air from infusion pumps and tubing, syringes and dialysis equipment. Devices that detect the presence of air in intravenous devices and notify the user could be used in order to stop the infusion when air is identified in the tubing. However, these devices also have limitations: the system could lead to a gap in patient treatment and does not prevent infusion of the air that is formed in the infusion line distal to the pump, as the health care provider explores to troubleshoot the system [20].

Oxygen should be administered as soon as possible to maximize oxygenation. The FiO_2_ should be adjusted to 100% if the patient is intubated. The reabsorption of the nitrogen from the bubble of air can be achieved via high-flow oxygen administration [21].

To displace the air embolus away from the right ventricular ejection tract, the patient should be placed in the left lateral decubitus and Trendelenburg position. In patients where this maneuver is insufficient to displace the air bubble, aspiration of air could be required trough a multiport central catheter or Swan Ganz catheter, but this often has limited value. This procedure has more chances of success if a catheter is inserted into the right atrium or ventricle before the procedure [21]. External cardiac compression could speed up the fragmentation and dispersion of large right ventricular air bubbles. Supportive care includes treatment of the shock, such as with volume resuscitation, vasopressors or inotropes [23].

In the presence of neurological deficits, hyperbaric oxygen therapy is indicated. Hyperbaric oxygen therapy represents the administration of 100% oxygen at a pressure above sea level and cause a reduction in the size of the air bubble, an increase in oxygen transfer to ischemic organs and a decrease in cerebral edema and gas volumes, enhances the partial pressure of dissolved oxygen and diminishes the activation of leukocytes [21,31]. Air emboli are composed of nitrogen (78%) and oxygen (21%). Oxygen is promptly metabolized and eliminated from circulation, and thus, hyperbaric oxygen therapy has the role of removing the remaining nitrogen, an objective achieved at high atmospheric pressures [32].

The pathophysiological effect of an air embolism is transient, and the impairment is reversible with proper treatment. There are no reports about long-term prognosis [26].

## 3. Fat Embolism

Fat embolism is a parenchymatous embolism defined as the release of fat emboli in the pulmonary and systemic circulation, in most cases of medullary origin, which affects the small vessels [29,33]. It is most often a complication of trauma or orthopedic surgery and can cause systemic reactions up to cardiorespiratory insufficiency and multiorgan failure. Pulmonary fat embolism (FE) caused by fat emboli was first described in 1862, when Zenker highlighted fat droplets in the small pulmonary arteries of a worker after a railway accident [18,34].

### 3.1. Incidence

Fat emboli are released into the circulation in 1–20% of trauma patients [22]. A proportion of 0.25–11% of patients with a single long-bone fracture develops fat embolism, but the percentage increases substantially in patients with multiple fractures. In patients suffering from pelvic or bilateral femoral fracture, the incidence increases to 4.8–33% [35,36].

Bone fractures in the lower extremities are the most common release sites for fat emboli. The fractures at the level of the upper extremities have a lower incidence. Very rare, in cases of trauma to the vertebrae or the rib cage, fat embolism can be diagnosed. It is also found in cases of those who undergo knee or hip replacement surgery [37].

### 3.2. Etiology

Although fat embolism occurs most frequently after bone fractures or recent trauma, other causes or predisposing factors should not be ignored either. Soft tissue injury, burns, decompression and septicemia are some of the causes encountered in clinical practice [34,38].

Activation of the coagulation system and vascular occlusion leads to bone marrow necrosis in patients with sickle cell disease. Bone marrow necrosis is a chronic condition in sickle cell disease, and during the vaso-occlusive crisis, the fat released from the marrow reaches the lungs or the systemic circulation. These mechanisms result in pulmonary damage and for approximately 33% of these patients, fat embolism is fatal [18,39]. A less common cause, but with clinical importance, is bone marrow injury through intramedullary endoscopy and intramedullary nail fixation [3,40]. In this case, when compared to that with the unreamed intramedullary nailing of diaphyseal femoral fractures, intramedullary nailing after reaming has been shown to be a safer procedure with a lower risk of systemic embolization [40]. There are case reports of pediatric patients who died after fat embolism caused by the insertion of the intraosseous catheter [41]. Cardiopulmonary resuscitation (CPR) is a well-known cause of fat embolism, due to bone injury, but also trauma to subcutaneous tissue [42].

The occurrence of fat embolism was observed after periurethral injection with bulking agents intended for the treatment of stress urinary incontinence [18,43]. In extremely rare situations, isolated cases where fat embolism appeared as a complication of pregnancy are reported [44]. Pregnant women with an increased risk of fat embolism are those with hemoglobinopathies and acute fatty liver. Moreover, Cesarean delivery increases the risk of fat embolism via the manipulation of subcutaneous abdominal cellular tissue and intraperitoneal fat tissue [44]. Other causes of FE could be represented by bone marrow harvesting or the mobilization of fat after liquefying hematoma [45].

It may also be seen in patients with hepatocellular cancer [46], fatty liver disease [47], acute pancreatitis with fat necrosis [48] and carbon tetrachloride poisoning [33].

In an era with a rising frequency of elective cosmetic and plastic surgery procedures, fat embolism may follow liposuction [49], gluteal augmentation with fat [50] and penile enlargement via autologous fat transfer [51]. Table 3 summarizes the causes of fat embolism.

### 3.3. Pathogenesis

The specific mechanisms of fat embolism are still unclear. However, there three are theories that try to explain the phenomena underlying its occurrence: the mechanical theory, the biochemical theory, and the coagulation theory.

The mechanical theory, first described in 1924, proposes that the fat cells originating from the site of the adipose tissue or bone marrow injury together with aggregated red blood cells and platelets block the pulmonary circulation, leading to local ischemia and organ failure [2,52]. In the presence of an arteriovenous shunt, systemic emboli can occur due to fat particles that reach the arterial circulation. Therefore, paradoxical cerebral embolism must be considered in cases of an altered mental status in trauma patients [18].

On the other hand, free fatty acids play an essential role in the pathophysiology of fat embolism. An increased content of free fatty acids was observed in the lungs of trauma patients, originating in the bone marrow [53]. Fat emboli cause activation of the inflammatory cascade and the biochemical response [54,55]. In the case of traumatized patients, increased serum lipase catalyzes the breakdown of fat drops from the lungs into free fatty acids and glycerol. The latter represent a local toxic factor. Endothelial damage occurs, and the presence of the inflammatory response promotes the agglutination of microemboli [56]. Thus, this theory does explain the occurrence of pulmonary symptoms 24–72 h after the trauma [18]. In cases of trauma, the associated proinflammatory status and increased concentrations of inflammatory markers (such as interleukin-6) increase the risk of fat embolism, endothelial cell damage and organ failure [56].

The coagulation therapy puts the inflammatory and prothrombotic response caused by circulating fat droplets in foreground. The activation of the coagulation cascade determines the growth of the thrombus and the associated vascular obstruction. Thus, thrombocytopenia and disseminated intravascular coagulation can be encountered [56].

### 3.4. Clinical Findings

In the case of fat embolism syndrome occurrence, several major and minor symptoms are encountered [33,57]. Respiratory distress, neurological manifestations that cannot be associated with head trauma and petechial rash distributed on the surface of the neck, anterior chest and mucous membranes are the major features included in FE syndrome [57].

A sudden cardiopulmonary collapse can be caused by a large embolus, but usually, the presentation of FE is represented by dyspnea, hypoxemia and tachypnea. Mechanical ventilation will be necessary in about half of the patients [58]. Hypoxemia is the clinical sign often found in cases of FE. Commonly, there is an insidious onset of tachypnea, dyspnea and hypoxia, which may progress to respiratory failure with the need for oxygen therapy [57]. The neurological changes that may occur are confusion, agitation, delirium, stupor, hemiplegia, quadriplegia, conduction aphasia, pupillary dilatation, blindness, convulsions and even coma, and these are usually reversible [33,57].

In 20–50% of cases, as part of the classic triad, a petechial rash may be present and spread to the oral mucous membranes, conjunctivae, the axillae, the head, the neck and the thorax. This distribution of petechiae is due to the fat droplets that accumulate in the aortic arch and further are led through the bloodstream to the non-dependent areas, irrigated by the carotid and subclavian arteries. These skin manifestations resolve in 5–7 days [57].

Gurd was the first to try to establish some criteria for the diagnosis of FE. In addition to the major diagnostic criteria (respiratory insufficiency, cerebral involvement and petechial rash), there are also minor features, non-specific, but frequently encountered in patients: tachycardia (>110/min), hypotension, fever (>38.5° Celsius), jaundice, anemia, thrombocytopenia, retinal petechiae, anuria, oliguria, elevated erythrocyte sedimentation rate, fat globules in urine or sputum [59]. Another two authors tried to establish some diagnostic criteria, including for example hypoxemia, acidosis or radiograph changes, but all of these criteria are based on a small series, and none have been validated in prospective studies [60,61]. These criteria are broad and have the possibility to capture some nonspecific cases of respiratory syndromes and extend unnaturally the incidence of FE [55].

### 3.5. Imaging Findings

There are no specific laboratory tests for the diagnosis of FE, but the following changes were observed: anemia, thrombocytopenia, increased inflammatory markers, hypocalcemia, decrease in free albumin [56].

The radiographic image can often be confused with post-traumatic pulmonary contusion. It is therefore important to differentiate the two entities. The pulmonary contusion is commonly asymmetric and unilateral and appears immediately after trauma; however, the radiographic image of fat embolism is evident after 24–48 h [29]. Non-specific bilateral infiltrates are noticed on a chest radiography [29,56]. Thoracic CT is the preferred imaging investigation in cases of fat embolism and the appearance of ground-glass opacities, and interlobar septal thickening with the pattern of “crazy paving” is described [62]. Nevertheless, this aspect is also non-specific, being also encountered in the case of ARDS, bacterial pneumonia, pulmonary oedema or lung hemorrhage [56]. Pulmonary ischemia can cause inflammation, microhemorrhage and edema, characterized by the appearance of centrilobular and interlobular nodules. In some cases, bone marrow or fat can be seen through the pulmonary parenchyma [29]. The severity of the disease is correlated with the degree of lung damage shown based on imaging findings [57].

For patients in which neurological symptoms develop, magnetic resonance imaging is recommended, being the most sensible method for cerebral embolism detection. T2-weighted images show small multiple non-confluent hyperintense lesions, described as a “starfield pattern”, in the subcortical, periventricular and deep white matter [55].

### 3.6. Treatment

Treatment in the case of FE is generally supportive, with the main goal of maintaining the patient’s oxygenation. Initially supplemental oxygen therapy is used, but some patients (10–44%) will need non-invasive or invasive ventilatory support [56]. Among the management strategies, we mention airway pressure release ventilation, extracorporeal membrane oxygenation and prone positioning. However, it must be emphasized that most patients are post-trauma, with multiple bone fractures, and thus, some of these supportive measures are difficult to institute [63].

There is recent evidence published on the use of extracorporeal membrane oxygenation (ECMO) in patients with severe pulmonary embolisms [64]. ECMO was used for 120 h in the postoperative period of a patient with multiple fractures of the ulna and femur, followed by a favorable patient evolution [63]. Another paper reported the preoperative initiation of venovenous ECMO (VV-ECMO) to prevent intraoperative fat embolism [65].

Neurological surveillance is mandatory. It may be considered for the prevention of seizures and may be necessary to improve cerebral perfusion pressure in cases of edema. In cases of cardiovascular instability, intravenous fluids can be used in association with inotropic drugs, peripheral constrictors and pulmonary vasodilators [56]. Drug treatment, such as heparin, aspirin, corticosteroids, N-acetylcysteine, hypertonic glucose or aliskiren, have been used over time, but without an obvious benefit to the patients [56,57].

Symptoms are often transient with good prognosis (mortality < 1.2%). Full recovery is expected under appropriate supportive therapy [66].

## 4. Amniotic Fluid Embolism

Amniotic fluid embolism (AFE) is a rare complication of pregnancy with an incidence of 1.9–6.1 per 100,000 births and a mortality rate of 60%, characterized by changes in oxygenation, hemodynamics and coagulation in the peripartum and postpartum period [67]. The condition is difficult to detect, and the diagnosis remains one of exclusion, and thus, the incidence is probably higher taking into consideration the underreported nonfatal cases [68]. The disease occurs typically during labor, after Caesarean or vaginal delivery or during second trimester evaluation procedures [69].

The mortality rate differs by the registry and is recorded between 37% in the British registry with 7% of the patients diagnosed with neurological impairment to 61% mortality in the United States with permanent neurologic deficits in 85% of the patients [69]. Mortality is related to the disease presentation and severity [70].

Some risk factors associated with amniotic fluid embolism have been reported, such as:

- Cesarean delivery with rates between 20–60% [71];
- Rupture of the membrane in 78% of the women in a United States registry, with early onset of symptoms [72];
- Maternal age around 33 years old [73];
- Black race [74];
- Multiparity;
- Medical induction of labor [75];
- Preexisting pathological conditions: placental abruption, placenta previa, eclampsia, uterine rupture, cervical lacerations [76];
- Forceps or vacuum delivery [74]

### 4.1. Physiopathology

The mechanism of AFE occurrence is poorly understood. It is presumed that amniotic fluid debris discovered in maternal circulation of patients with AFE are responsible for disease occurrence, via an unknown mechanism. Fetal squamous cells have been found in the blood flow of patients without a diagnosis of AFE, and this condition was diagnosed in patients without the presence of amniotic fluid debris. Animal studies to induce AFE upon the infusion of amniotic fluid directly into the vasculature of the animals fail to induce the disease [77]. In this context, there is a growing understanding that AFE may not be “embolic” in the conventional sense, the clinical manifestation being a result of a maternal immunologic response rather than the result of physical obstruction of pulmonary vessels [78]. It is postulated that when the elements contained in amniotic fluid (bradykinin, leukotriene, thromboxane, procoagulant factors and arachidonic metabolites) enter the maternal circulation, an immunologic response could be triggered, which is responsible for AFE manifestation [77].

### 4.2. Diagnosis

The diagnosis of AFE is clinical and is usually an exclusion diagnosis. The presentation could be various, starting from cardiopulmonary collapse with coagulopathy to minor or subclinical presentations [70].

Prodromal symptoms are sudden chills, sweating, shivering, coughing, nausea and vomiting [69]. The evolution of symptomatology could be divided in three phases. Initially, the patient presents with transient pulmonary and systemic hypertension with severe mismatch between ventilation and perfusion, hypoxemia and right heart failure. The second phase implicates left ventricular dysfunction that could lead to acute pulmonary edema. The final phase, the third, implies the presence of heart failure, acute respiratory distress syndrome or worsening of pulmonary injury and coagulopathy [70].

Respiratory symptoms manifest as bronchospasm, tachypnea and cyanosis, frequently culminating with pulmonary edema. Hypoxemias justify relentless, cyanosis, convulsions and coma. Cardiovascular collapse characterized by hypotension, tachycardia and arrhythmias may lead to cardiac arrest. The central nervous system is also involved, and the symptoms could be represented by an altered mental status, seizures, coma and death. Disseminated intravascular coagulopathy also occurs with severe bleeding [69].

There are some specific criteria for the diagnosis of AFE, divided into four categories: 1. respiratory distress, 2. cardiovascular collapse, 3. coagulopathy or disseminated intravascular coagulation and 4. seizures or coma. The presence of any of these criteria during labor, dilation and evacuation, Cesarean delivery or within 30 min peripartum is sufficient for the diagnosis in the absence of other diseases that explain the symptoms [68,78].

There are some tests that support the diagnosis and help with conducting the treatment. In order to detect the degree of hypoxemia, continuous pulse oximetry and arterial blood gas measurements should be achieved (usually there will be a low pH and PO_2_ and increased PCO_2_ and base excess levels). Prothrombin time is prolonged secondary to using clotting factors, and the fibrinogen level should be monitored (low levels should suggest intravascular disseminated coagulation), with the blood type, if a transfusion may be necessary. Serum tryptase and cardiac enzymes could be elevated. Decreased levels of complement (C3 and C4) have a sensitivity of 88% and a specificity of 100% for the diagnosis [79].

Chest radiography is nonspecific showing signs of acute pulmonary edema. The electrocardiogram may show findings that suggest right ventricular strain, such as ST segment and T wave changes. Transesophageal echocardiography together with pulmonary artery catheters may show elevated pulmonary artery pressure or right ventricular dysfunction and tricuspid regurgitation [79] The value of CT for the diagnosis of AFE is limited because of the fulminant evolution of this entity. The low number of cases where chest CT could be performed revealed diffuse bilateral ground-glass opacities [29].

Although historically the presence of fetal squamous cells in the pulmonary circulation was the diagnostic tool, because this type of cell is present in patients without symptoms of AFE, this test is considered today suggestive but not diagnostic for AFE syndrome, especially if the squamous cells are plentiful, are shielded by neutrophils or are accompanied by other fecal debris [79].

New markers are necessary for the diagnosis and understanding of the pathogenesis in AFE. Many biomarkers have been proposed for diagnosis purposes (TKH2- monoclonal antibody directed toward the sialosyl-Tn structure; STN—Sialosyl Tn and others), but further studies are necessary to determine their role in the diagnosis of this syndrome [70].

### 4.3. Treatment

Measures to prevent AFE should be established: trauma to the uterus must be avoided during rupture of membranes or insertion of a pressure catheter; incision of the placenta during Cesarean delivery should be avoided; excessively strong and frequent contractions must be controlled by beta adrenergic drugs or magnesium sulfate and oxytocic drugs must be used judiciously [79].

The treatment of AFE is supportive and should be focused on aggressive cardiovascular support, the management of coagulopathy and hemorrhage, treatment of hypoxia and delivery of the fetus if AFE appears before birth [79].

The treatment of coagulopathy could require massive transfusion, fresh frozen plasma, platelets and cryoprecipitate. There is some evidence suggesting that use of tranexamic acid could be efficient. Uterine atony should be treated with standard pharmacologic treatment. If the hemorrhage cannot be controlled, hysterectomy should be performed as soon as possible [80].

Despite improvements in the diagnosis and treatment of AFE, the mortality remains high and most of patients that survive will remain with neurologic deficits, kidney failure or cardiovascular diseases [79].

## 5. Septic Embolism

Septic pulmonary embolism (SPE) represents the embolization of a thrombus containing microorganisms (fungi, bacteria or parasites) into the lungs via pulmonary arteries [29]. This may be rare, but without proper treatment, it is associated with a high risk of mortality. SPE can imitate the thrombotic form, a reason why this should be considered in its differential diagnosis [36].

### 5.1. Etiology

The most common factors that can cause SPE are tricuspid valve endocarditis, skin infections, an immunocompromised state, infected central venous catheters, alcoholism, osteomyelitis [38] and intravenous drug abuse, but it is also associated with septic thrombophlebitis, post-anginal septicemia and periodontal disease [81]. Liver abscess, hemodialysis and pacemakers are also associated with septic pulmonary embolism [82]. Lemierre syndrome is an entity that can also cause septic pulmonary embolism, due to an anaerobic thrombophlebitis of the internal jugular vein [29].

### 5.2. Diagnosis

Because clinical and radiological findings are not specific, the diagnosis can be delayed. Signs and symptoms can vary from fever, hemoptysis, cough and shortness of breath in patients with an extra-pulmonary source of infection [1]. Clinical findings specific for the most common causes of SPE may not be present at the clinical exam of the patient. Cardiac murmurs can be absent in patients with tricuspid valve endocarditis, and clinical signs specific for Lemierre syndrome may be unnoticed unless the clinician is looking for inflammation at the anterior border of the sternocleidomastoid muscle [83]. The early diagnosis of SPE and differentiation from thromboembolic pulmonary embolism is essential since the treatment and prognosis is different [2].

Radiological findings are not specific as they can be seen both in thrombotic and non-thrombotic pulmonary embolism: hemidiaphragm elevation at the site of pulmonary artery occlusion, expansion of the central pulmonary artery, peripheral radiolucency due to diminished vascularity, pleural based wedged shaped areas of opacity due to infarction [38].

The gold standard modality of diagnosis of SPE is contrast-enhanced CT. It shows bilateral lung nodules, located mainly in the peripheral lung areas and in the lower lobes [5]. Nodules have different sizes and consolidation associated with pleural effusion [6]. CT may also show mediastinal or hilar lymphadenopathy, a dilated pulmonary branch (mycotic aneurysm) and a feeding vessel sign. The presence of a mycotic aneurysm has an important impact on the management of the patient because there is a high risk of rupture [38].

The feeding vessel sign represents a vessel leading directly to the nodule [84]. Some studies have found that a “halo sign” and “feeding vessel sign” are more frequent in SPE secondary to Gram-negative bacteria, whereas the cavitation and bronchograms are more common in Gram-positive bacterial embolisms [29]. The feeding vessel sign, which indicates the hematogenous nature of the nodules, is not specific for septic pulmonary embolism, as this image can be found in other diseases, such as bland pulmonary embolism and hematogenous metastasis [29], arteriovenous malformation, pulmonary vasculitis and pulmonary infarction [84]. Multi-detector CT is superior to the classical CT when we want to point up the feeding vessel sign [18]. When the pathogen is bacterial, there is a tendency for there to be more than one lesion, but when the lesion is located in one lobe or segment of the lung and the rest of it is normal, fungal infection should be taken into account [6]. The “halo sign”, which represents a central area of soft tissue attenuation surrounded by a halo of ground glass attenuation is a characteristic, yet non-specific, CT appearance for hemorrhagic nodules [18]. The differential diagnosis should be made with cavitary pulmonary metastases, granulomatosis with polyangiitis, rheumatoid lung nodules and tuberculosis [5].

An important objective is finding the primary source of infection, and extensive research needs to be performed: blood cultures, transthoracic and transesophageal echocardiography (for vegetation on the heart valve), contrast CT enhancement of the neck, chest, abdomen and pelvis, oral examination performed by a dentist and facial CT scans can identify an abscess in the infratemporal fossa (for periodontal disease) [85]. The bacteria involved in septic pulmonary embolism are particularly represented by Gram-positives, such as *Staphylococcus aureus*, followed by *Fusobacterium* species, *Eikenella*, *Porphyromonas*, streptococci and *Bacteroides*, but *Sphingomonas paucimobilis* and *Aeromonas* species can also cause SPE [3]. Patients who have immunological deficiencies, such as leukemia, lymphoma or other hematological neoplasias are affected by fungal emboli of *Candida*, *Aspergillus* and *Mucor* [3]. *Staphylococcus aureus* is most frequent in intravenous drug abusers, skin, soft tissue and catheter-related infection. *Candida* is also associated with catheter-related infections [82]. *Klebsiella pneumoniae* is the most common pathogen for liver abscess. *Fusobacterium* is common in Lemierre syndrome and oropharyngeal infections [82]. In patients with immunodeficiency, *Yersinia enterocolitica* should be considered as it can be complicated by abscesses and terminal ileitis [86]. Another rare cause of septic pulmonary embolism is represented by *Enterococcus granulosus*, due to the rupture of a cystic lesion into hepatic veins, in the right cardiac chamber or in the inferior vena cava [3].

### 5.3. Treatment

The treatment in SPE depends on the clinical presentation of the patient and the underlying cause. Empiric antibiotic therapy should be started, initially with glycopeptides and in appropriate settings, the addition of broad-spectrum antibiotics [87]. Depending on the results of the blood cultures, antibiotics can be modified and continued for at least 4–5 weeks. The time necessary for antibiotic therapy is guided by the clinical and para-clinical results [87], but there are no clear guidelines on the optimal duration of the therapy in SPE [88]. The removal of infected devices, cardiac surgery and tube drainage are indicated [81]. Patients with periodontal disease as a cause of SPE should receive tooth extraction and periodontal care [81]. Candidemia is the cause of 4% of SPEs and has a high mortality rate of approximately 40%, even when the patients receive proper antifungal treatments. The risk factors for candidemia include old age, broad-spectrum antibiotics, gastrointestinal surgery, central venous catheter and total parenteral nutrition. The international guidelines recommend 2 weeks of antifungal therapy after documented *Candida* spp from the bloodstream and resolution of the symptoms, but the proper duration of the intravenous therapy and step down to oral therapy has not been decided yet [89]. It is recommended that patients with risk factors for fungal infections receive antifungal drugs empirically to avoid poor prognosis [88].

Regarding the prognosis of the patients, in a study that included 20 patients with septic pulmonary embolism, the mortality rate was 30%. Non-survivors had higher serum creatinine and PaCO_2_ than the survivors and lower arterial pH. The most common complications for these patients were: acute respiratory failure, septic shock, acute kidney disease, acute respiratory distress syndrome and disseminated intravascular coagulation [90].

## 6. Tumor Embolism

Pulmonary tumor embolism (TE) represents the embolization of malignant cells into the pulmonary arteries. TE differs from metastatic lesions because the aggregated cells are intravascular and infrequently invade the parenchyma of the lung. Tumor cells are destroyed or remain latent inside the vascular lumen [3].

Based on postmortem evaluations, TE has an incidence of 2.4 to 26% [36]. The most common sources of TEs are represented by renal cell, gastric, breast, adrenocortical, choriocarcinoma, sarcomas and hepatocellular carcinomas [1].

**Figure 2:**
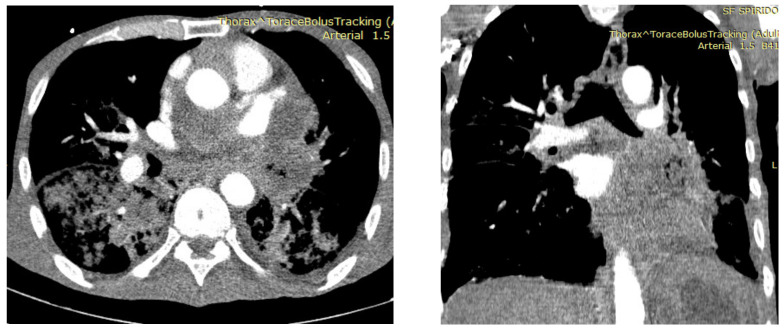
Tumoral infiltration of the mediastinum, with direct invasion and tumoral thrombosis of the left pulmonary artery and its lobar branches; partial tumoral thrombosis of the segmentary branches.

There are three categories of tumor embolisms:

- Type I also, called “true” tumor embolism, represents the embolization of the tumor through hematologic sending without invasions of the vessel walls;
- Type II results from a tumor developing into the pulmonary artery;
- Type III appears when a primary lung cancer or a metastatic tumor infiltrate and obstructs the pulmonary artery [1]. This aspect is illustrated in Figure 2.

The expansion of the TE ranges from embolization of the small vessels that may cause subacute pulmonary hypertension to occlusion of the main pulmonary artery mediated by a large volume of tumor material with acute signs of pulmonary embolization [91]. The risk of TE is higher after procedures that can promote fragmentation (e.g., surgical resection, chemotherapy or radiotherapy) and eventually circulation of the tumor mass into pulmonary circulation [4].

Tumor embolization is rarely large enough to cause demise. Usually, this will cause progressive dyspnea with progressively aggravation, cough, hemoptysis, pleuritic chest pain, fatigue and weight loss [4]. Physical examination exposes signs of right-sided ventricular overload and pulmonary hypertension [22]. Arterial blood gas analysis shows hypoxia, hypocapnia or an increased alveolar–arterial oxygen gradient [92].

In patients with neoplasia, it is difficult to distinguish between pulmonary thromboembolism and TE in terms of clinical and paraclinical findings. Therefore, in this category of patients, the presence of both thrombotic and a tumor embolism should be considered [18].

**Figure 3:**
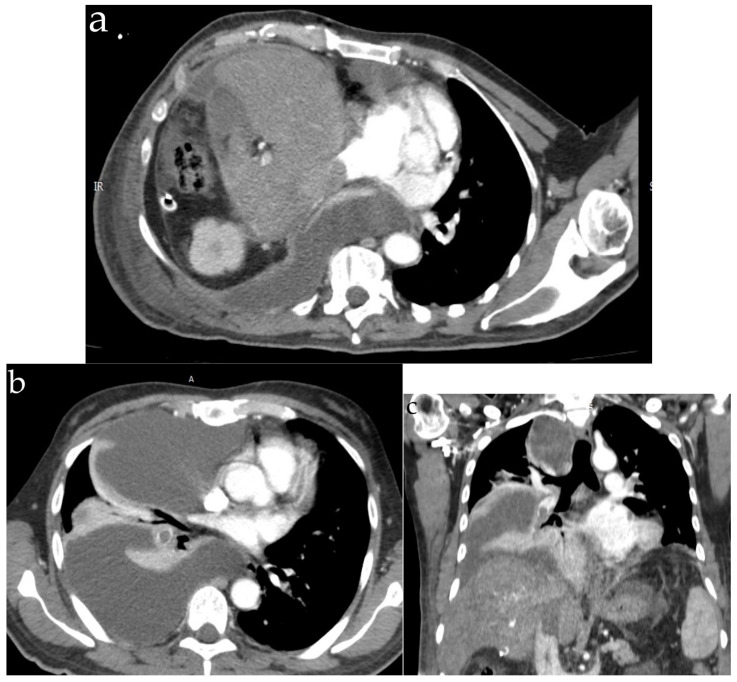
Thoraco-abdominal CT: hepatocellular carcinoma—local recurrence with enhancing tumoral thrombus into the inferior vena cava (**a**) and tumoral emboli introduction into the inferior right lobe branch of the pulmonary artery (**b**,**c**).

Tumor microembolism must be differentiated radiologically based on pneumonia, interstitial lung disease and tuberculosis [93]. On CT, the tumor embolism is difficult to distinguish from classic pulmonary embolism. The classic finding (when the centrilobular arteries are affected) is represented by an image of “tree-in-bud” formed by small centrilobular nodules of soft tissue attenuation, connected to other linear structures as branching originating from a single trunk [1]. An example of tumor embolism can be found in Figure 3. Other signs could be represented by dilated peripheral arteries, a specific tumoral component and no resolution despite anticoagulation treatment [2]. In patients where the main arteries are affected, the most common findings are filling defects that resemble acute or chronic pulmonary emboli [29].

An invasive technique for the diagnosis of TE is represented by cytological examination of blood aspirated from a wedged pulmonary artery catheter, which could detect the tumor cells. The sensitivity of this technique is 80% to 88%, and specificity is 82 to 94% [94].

Unfortunately, microscopic TE is hardly diagnosed before death because clinical, radiological and CT features of TE are similar to those of a pulmonary thromboembolism. A very recent study intended to determine the discrepancies between the antemortem diagnosis and autopsy results. The conclusion was that despite a significant improvement in radiological technologies over the past 30 years, the disparity between antemortem diagnosis and autopsy does not differ significantly [95].

There is no specific treatment available for TE. Specific therapeutic measures for the primary tumor associated with treatment for pulmonary hypertension are indicated [94].

## 7. Other Forms of Pulmonary Embolism

We will include in this section pulmonary embolism associated with the presence of foreign bodies in the pulmonary arteries, most frequently due to various medical procedures [2]. They are represented by:

- Catheter fragments, stents, guide wires;
- Dermal filler with silicone, hyaluronic acid or polyalkylimide [9];
- Cyanoacrylate and lipiodol used for gastric varices embolization [12];
- Polymethyl methacrylate cement from vertebroplasty or kyphoplasty;
- Talc, cellulose or starch injected intravenously by drug users;
- Mercury used very rarely in suicide attempts;
- Iodized oil embolization—patients who have undergone lymphangiography or transcatheter chemoembolization with iodized oil (alternative treatment of hepatocellular carcinoma);
- Liquid silicone used in cosmetic procedures;
- Radioactive pills used in the treatment of prostate cancer;
- Biosensors, devices implanted subcutaneously, used to generate, transmit and process biological signals, such as blood pressure, heart rate, respiratory signals or blood glucose levels [14].

## 8. Conclusions

Nontrombotic pulmonary embolisms are a group of diseases characterized by respiratory symptoms but with different etiologies, diagnosis possibilities and treatment strategies. The diagnosis of NTPE can be a formidable challenge, but the evidence published in medical literature is limited to case series. Knowledge of risk factors associated with NTPE could be a very good tool for the prevention or early diagnosis of this pathology. This paper has the purpose of increasing the awareness of nontrombotic pulmonary embolism as an overlooked cause of pulmonary hypertension and a cause of differential diagnosis with pulmonary thromboembolism.

## Figures and Tables

**Table 1:** Etiology of nontrombotic pulmonary embolism.

Cause	Biologic Products	Non-Biologic Material	Foreign Body
Iatrogenic (includes the effect of legal and illicit medical procedures)		Gas	Lost intravascular objects (catheters, stent, guide wire) [7]
		Cement [8]	Intraoperative materials [7]
		Dermal filler [9]	Radioactive seeds [10]
		Iodinated oil [7]	Ventriculoperitoneal shunts [11]
		Cyanoacrylate and lipiodol [12]	Yttrium-90 microsphere [7]
		Glue [13]	Biosensors [14]
Other (includes the following causes: trauma, pregnancy, infections, cancers, drug abuse, suicide)	Fat	Cotton [15]	Bullets [16]
	Amniotic fluid	Mercury [17]	
	Septic (bacteria, fungi)	Talc [10]	
	Tumor		
	Trophoblastic material [18]		
	Tissue (bone) [4]		
	Hydatid cyst [19]		

**Table 2:** Specific features for each type of NTPE.

Type of NTPE	Gas	Fat	Amniotic Fluid	Septic	Tumor
**Clinical features**	Respiratory distress in the context of a risk factor; any sudden respiratory, cardiovascular or neurologic symptoms after high-risk procedures	Respiratory distress (dyspnea, hypoxemia, tachypnea, hypoxia); neurological manifestations (confusion, agitation, delirium, stupor, hemiplegia, blindness, coma); petechial rash (on the mucous membranes, the axillae, the head, neck and thorax);	Respiratory distress; cardiovascular collapse; coagulopathy or disseminated intravascular coagulation; seizures or coma	Fever, hemoptysis, cough, shortness of breath in patients with an extra-pulmonary source of infection	Progressive dyspnea, cough, hemoptysis, pleuritic chest pain, fatigue and weight loss; signs of right-sided ventricular overload and pulmonary hypertension
**Lab tests**	Sudden decrease in end tidal CO_2_	Anemia, thrombocytopenia, elevated erythrocyte sedimentation rate, fat globules in urine or sputum, increased inflammatory markers, hypocalcemia, decrease in free albumin	Hypoxemia, prolonged prothrombin time, low fibrinogen levels, increased cardiac enzymes, low levels of complement (C3 and C4); the presence of fetal squamous cells in the pulmonary circulation	Blood cultures for detection of the primary infectious site	Hypoxia, hypocapnia or increased alveolar–arterial oxygen gradient
**Chest** **radiography**	Hyerlucency on pulmonary artery, right heart and systemic veins	Non-specific bilateral infiltrates	Non-specific acute pulmonary edema	Hemidiaphragm non-specific elevation at the site of pulmonary artery occlusion, expansion of the central pulmonary artery, peripheral radiolucency due to diminished vascularity, pleural based wedged-shaped areas of opacity due to infarction	
**Echocardiography**	Intracardiac air detection	Non-specific	Elevated pulmonary artery pressure, right ventricular dysfunction, tricuspid regurgitation	Vegetation on the heart valve	Non-specific
**Computer tomography**	Small amount of air in the main pulmonary artery, on the right heart or systemic veins; in severe cases, pulmonary hypertension and right heart strain are found	Ground-glass opacities, interlobar septal thickening with the pattern of “crazy paving”	Diffuse bilateral ground-glass opacities	Bilateral lung nodules, located mainly in the peripheral lung areas and in the lower lobes, mediastinal or hilar lymphadenopathy, a dilated pulmonary branch (mycotic aneurysm) and feeding vessel sign	The classic finding (affected centrilobular arteries) is an image of “tree-in-bud”; in cases where the main arteries are affected, the findings are filling defects that resemble acute or chronic pulmonary emboli

**Table 3:** Causes of nontrombotic pulmonary embolism.

Causes of Gas Embolism	Causes of Fat Embolism	Causes of Septic Embolism
*Invasive procedures:* placement, use and removal of catheter, contrast injection, pacemaker implantation, insufflation of air during endoscopy or endoscopic retrograde cholangiopancreatography, intra-aortic balloon rupture	*Trauma:* bone fractures, soft tissue injury, burns, cardiopulmonary resuscitation, decompression	*Infectious diseases:* tricuspid valve endocarditis, skin infections, osteomyelitis, infected central venous catheters, septic thrombophlebitis, post-anginal septicemia, periodontal disease, liver abscess, Lemierre syndrome
*Surgical interventions:* gynecological interventions, lung biopsy, laparoscopic abdominal insufflation, arthroscopy, prostatectomy, arthroscopy, carotid endarterectomy, intraoperative use of hydrogen peroxide, fibrinolytic pleural lavage	*Secondary to other diseases:* septicemia, sickle cell disease, hepatocellular cancer, fatty liver disease, acute pancreatitis, carbon tetrachloride poisoning, liquefying hematoma	*Diseases that predispose the patient to septic embolism:* immunocompromised state, alcoholism, intravenous drug abuse, hemodialysis, pacemakers
*Trauma:* chest trauma, barotrauma, ventilation-induced lung trauma	*Iatrogenic:* intraosseous catheter bulking agents injection, bone marrow harvest, cesarean delivery, intramedullary nail fixation, liposuction, gluteal augmentation, penile enlargement	
*Physiological conditions*: labor, delivery		

## Data Availability

Please contact the corresponding author for any supplemental data.

## References

[1] McCabe B.E., Veselis C.A., Goykhman I., Hochhold J., Eisenberg D., Son H. (2019). Beyond pulmonary embolism; nonthrombotic pulmonary embolism as diagnostic challenges. Curr. Probl. Diagn. Radiol..

[2] Khashper A., Discepola F., Kosiuk J., Qanadli S.D., Mesurolle B. (2012). Nonthrombotic pulmonary embolism. AJR Am. J. Roentgenol..

[3] Montagnana M., Cervellin G., Franchini M., Lippi G. (2011). Pathophysiology, clinics and diagnostics of non-thrombotic pulmonary embolism. J. Thromb. Thrombolysis.

[4] Bach A.G., Restrepo C.S., Abbas J., Villanueva A., Lorenzo Dus M.J., Schöpf R., Imanaka H., Lehmkuhl L., Tsang F.H., Saad F.F. (2013). Imaging of nonthrombotic pulmonary embolism: Biological materials, nonbiological materials, and foreign bodies. Eur. J. Radiol..

[5] Ufuk F., Kaya F., Sagtas E., Kupeli A. (2020). Non-thrombotic pulmonary embolism in emergency CT. Emerg Radiol..

[6] Bhalla S., Lopez-Costa I. (2007). MDCT of acute thrombotic and nonthrombotic pulmonary emboli. Eur. J. Radiol..

[7] Asah D., Raju S., Ghosh S., Mukhopadhyay S., Mehta A.C. (2018). Nonthrombotic Pulmonary Embolism from Inorganic Particulate Matter and Foreign Bodies. Chest.

[8] Patel Z., Sangani R., Lombard C. (2021). Cement pulmonary embolism after percutaneous kyphoplasty: An unusual culprit for non-thrombotic pulmonary embolism. Radiol. Case Rep..

[9] Boylan P.M., Santibañez M., Lounsbury N., Eltaki S.M. (2021). A nonthrombotic pulmonary embolus caused by polyalkylimide dermal filler: A case report and literature review of medication management. J. Am. Pharm. Assoc..

[10] Marschke G., Haber L., Feinberg M. (1975). Pulmonary talc embolization. Chest.

[11] Vernet O., Rilliet B. (2001). Late complications of ventriculoatrial or ventriculoperitoneal shunts. Lancet.

[12] Amin P., Pannu T., Mohamed R., Watson K. (2022). Nonthrombotic pulmonary embolism secondary to cyanoacrylate embolization of gastric varices. CMAJ.

[13] Michael P.G., Antoniades G., Staicu A., Seedat S. (2018). Pulmonary Glue Embolism: An unusual complication following endoscopic sclerotherapy for gastric varices. Sultan Qaboos Univ. Med. J..

[14] Carreira Villamor J.M., Flores Ríos E., Varela Ponte R. (2022). Iatrogenic Pulmonary Embolism. New Aspects. Arch. Bronconeumol..

[15] Kay J.M., Wilkins R.A. (1969). Cotton fibre embolism during angiography. Clin. Radiol..

[16] Fernandez-Ranvier G.G., Mehta P., Zaid U., Singh K., Barry M., Mahmoud A. (2013). Pulmonary artery bullet embolism-Case report and review. Int. J. Surg. Case Rep..

[17] Shi W., Jiao Y. (2020). Disseminated pulmonary emboli caused by mercury: A rare consequence of Munchausen’s syndrome. Clin. Case Rep..

[18] Jorens P.G., Van Marck E., Snoeckx A., Parizel P.M. (2009). Nonthrombotic pulmonary embolism. Eur. Respir. J..

[19] Aili A., Peng L., Zhang J., Liu Y., Peng L., Yi Q., Zhou H. (2021). Hydatid Pulmonary Embolism: A Case Report and Literature Review. Am. J. Case Rep..

[20] Brull S.J., Prielipp R.C. (2017). Vascular air embolism: A silent hazard to patient safety. J. Crit. Care.

[21] Cueto-Robledo G., Roldan-Valadez E., Mendoza-Lopez A.C., Palacios-Moguel P., Heredia-Arroyo A.L., Torres-Lopez I.D., Garcia-Cesar M., Torres-Rojas M.B. (2022). Air and Thrombotic Venous Embolism in a Department of Emergency Medicine. A Literature Review. Curr. Probl. Cardiol..

[22] Rossi S.E., Goodman P.C., Franquet T. (2000). Nonthrombotic pulmonary emboli. AJR Am. J. Roentgenol..

[23] Lanfranco J., Romero Legro I., Freire A.X., Nearing K., Ratnakant S. (2017). Pulmonary Air Embolism: An Infrequent Complication in the Radiology Suite. Am. J. Case Rep..

[24] Wong S.S., Kwaan H.C., Ing T.S. (2017). Venous air embolism related to the use of central catheters revisited: With emphasis on dialysis catheters. Clin. Kidney J..

[25] Buckridge N., Frisch S., Sinert R. (2021). Iatrogenic Pulmonary Air Embolism with Rapid Resolution: A Case Report. J. Emerg. Med..

[26] Xiao P.X., Hu Z.Y., Zhang H., Pan C., Duan B.X., Chen S.L. (2013). Massive pulmonary air embolism during the implantation of pacemaker, case reports and literature analysis. Eur. Rev. Med. Pharmacol. Sci..

[27] Maqsood M.H., Mirza N., Hanif M.A., Hanif H., Saleem M., Maqsood M.A., Fatima I., Tahir M.M. (2019). Clinical Presentation, Diagnosis, and Management of Air Embolism During Endoscopic Retrograde Cholangiopancreatography. Gastroenterol. Res..

[28] Uysal E., Alkan N., Cam B. (2019). A life-threatening condition: The pulmonary artery air embolism. Turk. J. Emerg. Med..

[29] Pena E., Dennie C., Franquet T., Milroy C. (2012). Nonthrombotic Pulmonary Embolism: A Radiological Perspective. Semin. Ultrasound CT MRI.

[30] Schafer S.T., Lindemann J., Brendt P., Kaiser G., Peters J. (2008). Intracardiac transvenous echocardiography is superior to both precordial Doppler and transesophageal echocardiography techniques for detecting venous air embolism and catheter-guided air aspiration. Anesth. Analg..

[31] Tovar E.A., Del Campo C., Borsari A., Webb R.P., Dell J.R., Weinstein P.B. (1995). Postoperative management of cerebral air embolism: Gas physiology for surgeons. Ann. Thorac. Surg..

[32] Malik N., Claus P.L., Illman J.E., Kligerman S.J., Moynagh M.R., Levin D.L., Woodrum D.A., Arani A., Arunachalam S.P., Araoz P.A. (2017). Air embolism: Diagnosis and management. Future Cardiol..

[33] Milroy C.M., Parai J.L. (2019). Fat Embolism, Fat embolism syndrome and the autopsy. Acad. Forensic. Pathol..

[34] Gresham G.A. (1986). Fat embolism. Forensic Sci. Int..

[35] Kontakis G.M., Tossounidis T., Weiss K., Pape H.C., Giannoudis P.V. (2006). Fat embolism: Special situations bilateral femoral fractures and pathologic femoral fractures. Injury.

[36] Gittens J.T., Semionov A., Pressacco J. (2018). Imaging of Pulmonary Embolus: Thrombotic, Nonthrombotic and Mimickers. Can. Assoc. Radiol. J..

[37] Koessler M.J., Fabiani R., Hamer H., Pitto R.P. (2001). The clinical relevance of embolic events detected by transesophageal echocardiography during cemented total hip arthroplasty: A randomized clinical trial. Anesth. Analg..

[38] Unal E., Balci S., Atceken Z., Akpinar E., Ariyurek O.M. (2017). Nonthrombotic Pulmonary Artery Embolism: Imaging Findings and Review of the Literature. Am. J. Roentgenol..

[39] Tsitsikas D.A., Bristowe J., Abukar J. (2020). Fat Embolism Syndrome in Sickle Cell Disease. J. Clin. Med..

[40] Hogel F., Gerlach U.V., Sudkamp N.P., Muller C.A. (2010). Pulmonary fat embolism after reamed and unreamed nailing of femoral fractures. Inj. Int. J. Care Inj..

[41] Castiglioni C., Carminati A., Fracasso T. (2022). Fat embolism after intraosseous catheters in pediatric forensic autopsies. Int. J. Legal Med..

[42] Ondruschka B., Baier C., Bernhard M., Buschmann C., Dredler J., Schote J., Zwirner J., Hammer N. (2019). Frequency and intensity of pulmonary bone marrow and fat embolism due to manual or automated chest compressions during cardiopulmonary resuscitation. Forensic Sci. Med. Pathol..

[43] Currie I., Drutz H.P., Deck J., Oxorn D. (1997). Adipose tissue and lipid droplet embolism following periurethral injection of autologous fat: Case report and review of the literature. Int. Urogynecol. J. Pelvic Floor Dysfunct..

[44] Schrufer-Poland T., Singh P., Jodicke C., Reynolds S., Maulik D. (2015). Nontraumatic fat embolism found following maternal death after Cesarean delivery. Am. J. Perinatol. Rep..

[45] Baselga J., Reich L., Doherty M., Gulati S. (1991). Fat embolism syndrome following bone marrow harvesting. Bone Marrow Transpl..

[46] Sakashita M., Sakashita S., Sakata A., Uesugi N., Ishige K., Hyodo I., Nouguchi M. (2017). An autopsy case of non-traumatic fat embolism syndrome. Pathol. Int..

[47] Schulz F., Trubner K., Hilderbrand E. (1996). Fatal fat embolism in acute hepatic necrosis with associated fatty liver. Am. J. Forensic Med. Pathol..

[48] Guardia S.N., Bilbao J.M., Murray D., Warren R.E., Sweet J. (1989). Fat embolism in acute pancreatitis. Arch. Pathol. Lab. Med..

[49] Cantu C.A., Pavlisko E.N. (2018). Liposuction-Induced Fat Embolism Syndrome: A Brief Review and Postmortem Diagnostic Approach. Arch. Pathol. Lab. Med..

[50] Pena W., Cardenas-Camarena L., Bayter-Marin J.E., McCormick M., Duran H., Ramos-Gallardo G., Robles-Cervantes J.A., Macias A.A. (2019). Macro Fat Embolism After Gluteal Augmentation with Fat: First Survival Case Report. Aesthet. Surg. J..

[51] Zilg B., Rasten-Almqvist P. (2017). Fatal Fat Embolism After Penis Enlargement by Autologous Fat Transfer: A Case Report and Review of the Literature. J. Forensic Sci..

[52] Yunle M., Zhang M., Ling H., Huang S., Miao Q., Yu Y., Zhang F., Qiu P., Li D. (2020). Nontraumatic Multiple-Organ Fat Embolism An Autopsy Case and Review of Literature. Am. J. Forensic Med. Pathol..

[53] Baker P.L., Pazell J.A., Peltier L.F. (1971). Free fatty acids, catecholamines, and arterial hypoxia in patients with fat embolism. J. Trauma.

[54] Mastrangelo A.M., Jeitner T.M., Eaton J.W. (1998). Oleic acid increases cell surface expression and activity of CD11b on human neutrophils. J. Immunol..

[55] Timon C., Keady C., Murphy C.G. (2021). Fat Embolism Syndrome—A Qualitative Review of its Incidence, Presentation, Pathogenesis and Management. Malays. Orthop. J..

[56] Luff D., Hewson D.W. (2021). Fat embolism syndrome. BJA Educ..

[57] Rothberg D.L., Makarewich C.A. (2018). Fat Embolism and Fat Embolism Syndrome. J. Am. Acad. Orthop. Surg..

[58] King M.B., Harmon K.R. (1994). Unusual forms of pulmonary embolism. Clin. Chest Med..

[59] Gurd A.R. (1970). Fat embolism: An aid to diagnosis. J. Bone Joint Surg. Br..

[60] Lindeque B.G., Schoeman H.S., Dommisse G.F., Boeyens M.C., Vlok A.L. (1987). Fat embolism and the fat embolism syndrome. Adouble-blind therapeutic study. Br. J. Bone Jt. Surg..

[61] Mellor A., Soni N. (2001). Fat embolism. Anaesthesia.

[62] Ong S.C.L., Balasingam V. (2017). Characteristic imaging findings in pulmonary fat embolism syndrome (FES). BMJ Case Rep..

[63] Webb D.P., McKamie W.A., Pietsch J.B. (2004). Resuscitation of fat embolism syndrome with extracorporeal membrane oxygenation. J. Extra Corpor. Technol..

[64] Lari A., Abdulshakoor A., Zogheib E., Assaf N., Mojallal A., Lari A.R., Bauer C., Sinna R. (2020). How to save a life from macroscopic fat embolism: A narrative review of treatment options. Aesthet. Surg. J..

[65] Popovich I., Singh V., Vickery B. (2019). Perioperative support of a patient with fat embolism syndrome with extracorporeal membrane oxygenation. BMJ Case Rep..

[66] Li S., Zou D., Qin Z., Liu N., Zhang J., Li Z., Shao Y., Deng K., Chen Y., Huang P. (2015). Nonfracture-Associated Pulmonary Fat Embolism After Blunt Force Fatality: Case Report and Review of the Literature. Am. J. Forensic Med. Pathol..

[67] Clark S.L. (2014). Amniotic fluid embolism. Obstet. Gynecol..

[68] Stafford I., Sheffield J. (2007). Amniotic fluid embolism. Obstet. Gynecol. Clin. N. Am..

[69] Rudra A., Chatterjee S., Sengupta S., Nandi B., Mitra J. (2009). Amniotic fluid embolism. Indian J. Crit. Care Med..

[70] Sultan P., Seligman K., Carvalho B. (2016). Amniotic fluid embolism: Update and review. Curr. Opin. Anaesthesiol..

[71] Lau G., Chui P.P. (1994). Amniotic fluid embolism: A review of 10 fatal cases. Singap. Med. J..

[72] Clark S.L., Hankins G.D.V., Dudley D.A., Porter T.F. (1995). Amniotic fluid embolism: Analysis of the national registery. Am. J. Obstet. Gynecol..

[73] Gilbert W.M., Danielson B. (1999). Amniotic fluid embolism: Decreased mortality in a populationbased study. Obstet. Gynecol..

[74] Abenhaim H.A., Azoulay L., Kramer M.S., Leduc L. (2008). Incidence and risk factors of amniotic fluid embolisms: A population-based study on 3 million births in the United States. Am. J. Obstet. Gynecol..

[75] Gei G., Hankins G.D. (2000). Amniotic fluid embolism: An update. Contemp. Ob Gyn.

[76] Kramer M.S., Rouleau J., Baskett T.F., Joseph K.S. (2006). Amniotic-fluid embolism and medical induction of labour: A retrospective, population-based cohort study. Lancet.

[77] Brennan M.C., Moore L.E. (2013). Pulmonary embolism and amniotic fluid embolism in pregnancy. Obstet. Gynecol. Clin. N. Am..

[78] Benson M.D. (2014). Amniotic fluid embolism: The known and not known. Obstet. Med..

[79] Kaur K., Bhardwaj M., Kumar P., Singhal S., Singh T., Hooda S. (2016). Amniotic fluid embolism. J. Anaesthesiol. Clin. Pharmacol..

[80] Pacheco L.D., Clark S.L., Klassen M., Hankins G.D.V. (2020). Amniotic fluid embolism: Principles of early clinical management. Am. J. Obstet. Gynecol..

[81] Hatani T., Takemura M., Inoue D., Takamatsu K., Ishitoko M., Itotani R., Suzuki S., Matsumoto M., Sakuramoto M., Fukui M. (2013). Septic pulmonary embolism due to periodontal disease: Septic embolism due to periodontitis. Respirology.

[82] Ye R., Zhao L., Wang C., Wu X., Yan H. (2014). Clinical characteristics of septic pulmonary embolism in adults: A systematic review. Respir. Med..

[83] Fred H.L., Harle T.S. (1969). Septic Pulmonary Embolism. Dis. Chest.

[84] Tale S., Ghosh S., Pahel Meitei S., Kolli M., Garbhapu A.K. (2021). Feeding vessel sign: A radiological sign of septic pulmonary embolism. QJM Int. J. Med..

[85] Watanabe T., Yokoe M., Noguchi Y. (2019). Septic pulmonary embolism associated with periodontal disease: A case report and literature review. BMC Infect. Dis..

[86] Dejima A., Yamamoto N., Hasatani K. (2021). Yersinia enterocolitica infection with septic pulmonary embolism and liver and intestinal lymph node abscesses. BMJ Case Rep..

[87] Goswami U., Brenes J.A., Punjabi G.V., LeClaire M.M., Williams D.N. (2014). Associations and Outcomes of Septic Pulmonary Embolism. Open Respir. Med. J..

[88] Jiang J., Liang Q., Liu L., Cai S., Du Z., Kong J., Chen Y. (2019). Septic pulmonary embolism in China: Clinical features and analysis of prognostic factors for mortality in 98 cases. BMC Infect. Dis..

[89] Okuno D., Oshima K., Miyazaki T., Ashizawa N., Hirayama T., Takazono T., Saijo T., Yamamoto K., Imamura Y., Yamaguchi H. (2021). Duration of antifungal therapy for septic pulmonary embolism caused by Candida albicans from a central venous catheter: A case report. Clin. Case Rep..

[90] Chou D.W., Wu S.L., Chung K.M., Han S.C., Cheung B.M.H. (2016). Septic Pulmonary Embolism Requiring Critical Care: Clinicoradiological Spectrum, Causative Pathogens and Outcomes. Clinics.

[91] Huang H.K., Huang C.H., Wu C.C., Chen W.J. (2011). An unusual cause of acute pulmonary embolism. Int. J. Cardiol..

[92] Chan C.K., Hutcheon M.A., Hyland R.H., Smith G.W., Patterson B.J., Matthay R.A. (1987). Pulmonary tumor embolism: A critical review of clinical, imaging, and hemodynamic features. J. Thorac. Imaging.

[93] Konstantinides S.V., Meyer G., Becattini C., Bueno H., Geersing G.J., Harjola V.P., Huisman M.V., Humbert M., Jennings C.S., Jiménez D. (2020). ESC Scientific Document Group. 2019 ESC Guidelines for the diagnosis and management of acute pulmonary embolism developed in collaboration with the European Respiratory Society (ERS). Eur. Heart J..

[94] Rajdev K., Madan U., McMillan S., Wilson K., Fisher K., Hein A., Patil A., Bista S., Hershberger D., Boer B. (2022). Pulmonary Tumor Embolism and Pulmonary Tumor Thrombotic Microangiopathy Causing Rapidly Progressive Respiratory Failure: A Case Series. J. Investig. Med. High Impact. Case Rep..

[95] He X., Anthony D.C., Catoni Z., Cao W. (2021). Pulmonary tumor embolism: A retrospective study over a 30-year period. PLoS ONE.

